# A Rare Case of Herpes Simplex Virus and Cytomegalovirus Dual Infection Inducing Unremitting Ulcerative Colitis

**DOI:** 10.7759/cureus.45166

**Published:** 2023-09-13

**Authors:** Rivers A Hock, Mohammad Yousaf, Jesse C Allen, Ethan Heh, Mark Raynor, Osvaldo Padilla, Diego P Peralta

**Affiliations:** 1 Internal Medicine, Paul L. Foster School of Medicine, Texas Tech University Health Sciences Center, El Paso, USA; 2 Pathology, Paul L. Foster School of Medicine, Texas Tech University Health Sciences Center, El Paso, USA; 3 Infectious Diseases, Texas Tech University Health Sciences Center, El Paso, USA

**Keywords:** immunosuppression complications, inflammatory bowel disease, herpes simplex virus, cytomegalovirus, ulcerative colitis

## Abstract

Ulcerative colitis (UC) is a subtype of inflammatory bowel disease that results in inflammation and ulceration in the lining of the large intestine. Patients with UC are frequently prescribed immunosuppressive medications to treat their symptoms, resulting in an increased risk of reactivation of many latent viruses, including herpes simplex virus (HSV) and cytomegalovirus (CMV). However, it is rare for a patient to present with simultaneous reactivation of both viruses. Here, we document the presentation, hospital course, and clinical findings of a UC patient with HSV and CMV dual infection. We also describe treatment strategies and prophylactic measures for managing a dual infection. This is seen through initiating valganciclovir in the outpatient setting following the diagnosis.

## Introduction

Ulcerative colitis (UC) is a chronic idiopathic inflammatory bowel disease (IBD) that primarily affects the colon and rectum, resulting in continuous mucosal inflammation and ulceration, typically occurring in a recurring and relapsing manner [1]. It classically presents with bloody diarrhea with or without mucus, rectal urgency, tenesmus, and abdominal pain often relieved by defecation [2]. Treatment is typically aimed at targeting histologic remission (i.e., mucosal healing), and mesalamine is the first-line therapy for induction of remission in mild disease [3]. If it is unsuccessful, systemic corticosteroids can be added. When ulcerative colitis becomes moderate-to-severe, the introduction of biologics such as infliximab, adalimumab, vedolizumab, ustekinumab, and the Janus kinase (JAK) inhibitor tofacitinib can be used [4,5].

Cytomegalovirus (CMV) is a member of the Herpesviridae family, which commonly causes asymptomatic infections within immunocompetent individuals before transitioning into a lifelong dormant phase [6]. CMV is then known to reactivate within immunocompromised patients and eventually cause end-organ damage [7]. A refractory response to steroid therapy is a well-known phenomenon in cases of CMV colitis, and recent guidelines suggest that CMV infection should be considered in cases of severe UC [8,9]. CMV diagnosis through viral DNA detection via PCR or immunohistochemical staining of a colonic biopsy sample is preferred over serology [10-13].

Herpes simplex virus (HSV-1 or HSV-2) infection is less commonly reported in IBD patients and has a less clear role in UC flares [14]. However, chronic inflammation and immunosuppressive agents such as corticosteroids, azathioprine, and mesalamine are known to cause increased infection risk [15].

Current evidence suggests that antiviral therapy in patients with active CMV and HSV infection results in higher clinical remission rates in UC patients that are steroid-dependent or steroid-refractory [16,17]. Here, we present the clinical course of a patient with a relapsing acute on chronic flare of UC found to be complicated by CMV and HSV dual infection.

## Case presentation

A 23-year-old African American man with ulcerative colitis (UC) presented to the emergency department (ED) on the recommendation from his outpatient GI physician to receive a blood transfusion for a low hemoglobin value of 5.5 g/dL that lasted for one week. UC was initially diagnosed seven months before his presentation via colonoscopy. On presentation, the patient described multiple instances of hematochezia, dizziness, fatigue, and intermittent nausea. He had suffered from persistent diarrhea for the last year, with 10-20 watery bowel movements per day. He was prescribed monthly vedolizumab and received four infusions, but he missed an appointment the month before the ED presentation. The patient took prednisone for flares with iron supplements, mesalamine (for six months), and azathioprine (for seven months) for his UC complaints. The patient was afebrile and hemodynamically stable in the ED. A complete physical exam revealed a thin pale male with concerns of malnourishment and no other abnormalities. Initial bloodwork showed low hemoglobin, ferritin, iron level, and transferrin saturation, supporting iron deficiency anemia (Table 1).

**Table 1:** Baseline and posttransfusion bloodwork results *Abnormal lab value; NP: Not performed

Tests	Normal Range	Before Transfusion	After Transfusion
White blood cells	4.5-11.0 x 10^3^/µL	7.97 x 10^3^/µL	7.03 x 10^3^/µL
Hemoglobin	12.0-15.0 g/dL	5.3 g/dL*	8.3 g/dL*
Hematocrit	40 to 54%	22.6%*	28.5%*
Mean corpuscular volume	82-98 fL	50.9 fL*	62.2 fL*
Platelets	150-450 x 10^3^/uL	586 x 10^3^/uL*	327 x 10^3^/µL
Sodium, serum	135-145 mmol/L	134 mmol/L*	134 mmol/L*
Potassium, serum	3.5-5.1 mmol/L	4.0 mmol/L	3.8 mmol/L
Chloride, serum	98-107 mmol/L	100 mmol/L	104 mmol/L
Glucose	74-106 mg/dL	87 mg/dL	79 mg/dL
Creatinine	0.52-1.04 mg/dL	0.76 mg/dL	0.70 mg/dL
Blood urea nitrogen	7-17 mg/dL	14 mg/dL	8 mg/dL
Ferritin	24-336 ng/mL	3.11 ng/mL*	NP
Iron, serum	49-181 µg/dL	16 µg/dL*	NP
Transferrin	206-381 mg/dL	282 mg/dL	NP
Transferrin saturation	19-39%	5%*	NP
Total iron binding capacity	261-462 µg/dL	345 µg/dL	NP

**Figure 1:**
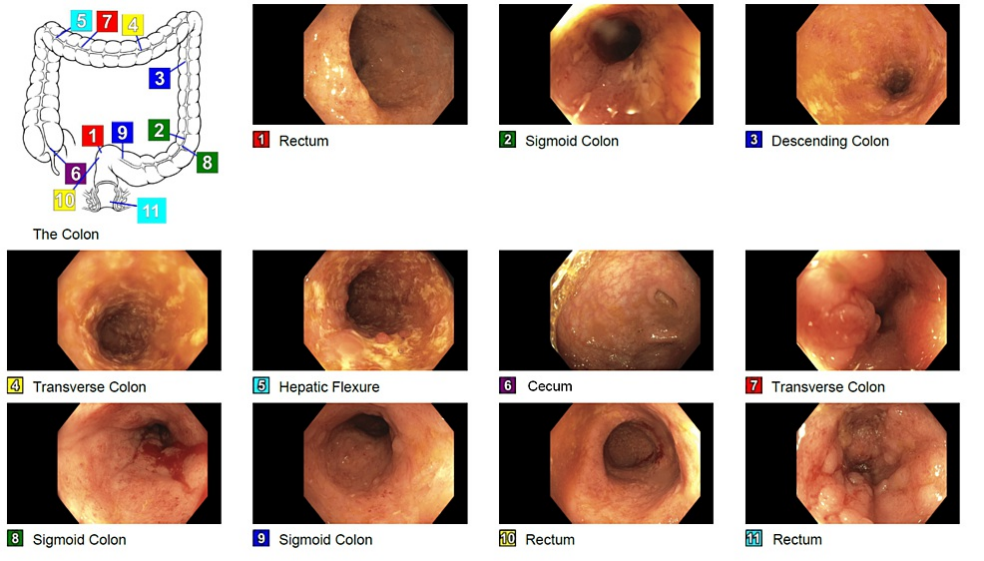
Colonoscopy findings Mild (Mayo Score 1) ulcerative colitis in the cecum. Moderately active (Mayo Score 2) ulcerative colitis from the rectum to the ascending colon with scattered pseudopolyps and segmental narrowing in the sigmoid, descending, and transverse colon.

**Figure 2:**
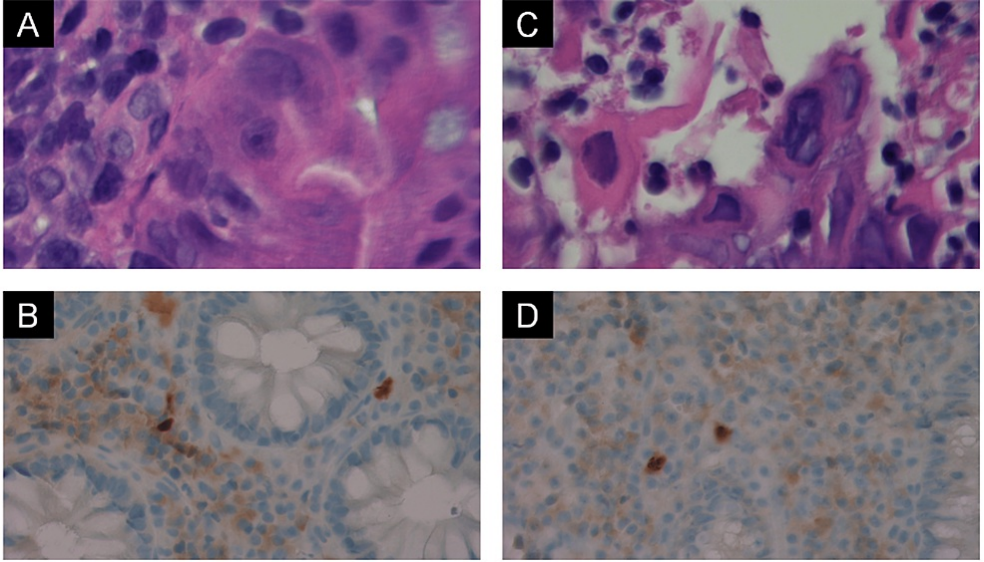
Biopsy images A: A rare epithelial cell is shown by H&E at 40X magnification with an enlarged nucleoli-like structure (center cell), which is suggestive of a CMV nuclear inclusion. B: A CMV immunohistochemical stain shows some nuclear staining in some cells. C: An epithelial cell is shown by H&E at 40X magnification with a “ground glass” nuclear appearance and an adjacent multinucleated cell on the right, which are cytologic features seen with Herpes-infected epithelial cells. D: An immunohistochemical stain for HSV 1/2 is performed, which shows occasional cytoplasmic-positive cells.

The patient was admitted and received two units of packed red blood cells. He was considered for an acute or chronic flare of UC, and the gastroenterology (GI) team was consulted. Stool workup, including culture, ova and parasite, and Clostridioides difficileantigen and toxins, were all ordered and returned negative. A colonoscopy revealed continuous and circumferential inflammation, moderately active UC, from the rectum to the ascending colon, and a narrowing of the sigmoid (Figure 1). Biopsy immunohistochemistry was positive for CMV in the cecum and ascending, transverse, and descending colon. HSV was positive in the ascending and descending colon and rectum (Figure 2).

Following colonoscopy, GI recommended starting intravenous (IV) methylprednisone for five days, followed by a tapering prednisone regimen. On day five, due to decreased blood in stool and clinical stability, the patient was discharged to continue prednisone oral prescription in addition to his home medications and was scheduled for follow-up at the outpatient GI clinic. 

Biopsy immunohistochemistry was positive for CMV in the cecum and ascending, transverse, and descending colon. HSV was positive in the ascending and descending colon and rectum (Figure 2). Results were confirmed after discharge. 

At GI follow-up one month after discharge, the patient had fully completed his steroid taper but had not reported for vedolizumab infusion. He was started on oral valganciclovir 900 mg twice daily at this time for dual viral colitis. Serum CMV and HSV PCR tests were ordered, and he was referred to the infectious diseases (ID) clinic for further management. PCR testing came back negative for both CMV and HSV. During his ID clinic visit, the patient had completed a four-week course of high-dose valganciclovir and was transitioned to daily low-dose prophylaxis, 900 mg. On the latest visit, five months post-discharge, the patient reported loose stool and small amounts of rectal bleeding. The patient discontinued his vedolizumab due to a loss of follow-up with GI. The patient continues with his azathioprine but has stopped taking prednisone.

## Discussion

Based on an extensive literature search, this is only one of the very few reports of CMV and HSV coinfection in a UC patient. Of the relevant results found from a PubMed and Google Scholar search, there were only three articles and a total of five patients with reported CMV and HSV dual colitis positivity. These are reported in Table 2. Our case differs in terms of immunosuppressive regimen prior to infection and clinical context.

**Table 2:** CMV and HSV dual infection in patients with ulcerative colitis reported in PubMed and Google Scholar databases

Author and Year	Study Type	Age and Sex	Patient Characteristics	Treatment	Outcome
Leal et al., 2022 [18]	Case report	63-year-old female	Ulcerative colitis treated with corticosteroids for 2 months developed respiratory infections, diabetes, and perianal ulcers	Ganciclovir, acyclovir, valganciclovir, and eventual total proctocolectomy and ileostomy	Asymptomatic 2 months later
Banerjee et al., 2009 [19]	Prospective cross-sectional study	No details were reported. Age range 14 to 61 years. Included male and female patients	Three out of 49 patients diagnosed with CMV/HSV colitis UC for more than 6 months were treated with systemic corticosteroids prior	Mesalamine, no antiviral therapy	Achieved remission
Huang et al., 2022 [20]	Case report	46-year-old male	HIV/AIDS nonadherent to antiretroviral therapy; 2 months of worsening non-bloody diarrhea esophagitis, gastritis, and severe colitis bowel perforation during treatment	Valacyclovir and ganciclovir	Lost to follow-up

Many researchers have theorized that immunomodulators and corticosteroids given on long-term UC management contribute directly to the reactivation of latent HSV and CMV [21,22]. Reactivation is considered to lead directly to refractory treatment response to corticosteroids and to worsen flares. The presence of these clinical features necessitates investigation for possible CMV infection within current guidelines. In vitro studies of THP-1 cells have demonstrated that reactivated CMV upregulates glucocorticoid receptors (GR), increases the ratio of GRβ/α, and increases the phosphorylation of GRα. Additionally, CMV lytic infection contributes to UC flares by directly increasing the pro-inflammatory cytokines IL-6 and TNF-α while decreasing the anti-inflammatory cytokine IL-5 [23]. 

Numerous cohort studies support these findings. In an Italian cohort study, 36% of steroid-resistant Crohn's and UC patients with acute colitis were found to be CMV-positive [24]. Additionally, a Korean study detected CMV infection in 43% and 67% of patients with moderate-to-severe active and steroid-refractory UC [25]. HSV involvement in the GI tract is a rare phenomenon, almost always found in immunosuppressed patients, and is associated with a significant increase in mortality [26,27]. A Japanese study powered to look for colitis co-infection among 81 patients treated for miscellaneous causes of colitis investigated incidences of EBV, CMV, HHV-6, HHV-7, parvovirus B19, and HSV-1. They found zero instances of HSV-1 colitis. The combination of EBV and CMV was the most significantly reported co-infection in these patients [28].

The first step in treatment for this patient was high-dose IV methylprednisone and a tapered prednisone regimen. This is meant to induce remission of the current acute inflammatory process in an attempt to stabilize the patient. Inflammation is known to trigger both CMV and HSV reactivation, making immediate antiviral therapy futile until the current UC flare is controlled [15,29]. After calming an acute flare, antiviral therapy has been shown to reverse steroid resistance and improve surgery-free survival [30-32]. Antivirals are also effective and recommended for prophylaxis of recurrent CMV infection. Recent studies show that 77.8% of UC patients on antiviral therapy-maintained remission for 12 months compared to 45% who were not treated. Additionally, 77.8% maintained 12-month remission in cases of steroid-refractory UC, whereas only 19.4% did so in untreated patients [17].

The standard of care for CMV colitis is either oral valganciclovir or intravenous ganciclovir, depending on the patient's clinical status [33]. Valganciclovir is known to have a slightly higher risk for neutropenia (8.2% vs. 3.2% from ganciclovir), with both drugs having a similar overall safety profile.

A three-week course of valganciclovir 450 mg twice daily has been shown to dramatically improve active symptoms of CMV colitis both clinically and endoscopically [34]. Furthermore, a once-daily oral valganciclovir has been found to be as clinically effective and well-tolerated as oral ganciclovir three times daily for CMV prophylaxis [35].

Regarding HSV colitis, valganciclovir is known to show coverage across the entire Herpesviridae family [36,37]. Acyclovir is occasionally considered in cases of HSV colitis [18]. However, prescribing multiple antivirals can often lead to undesirable side effects. Compared to acyclovir, valganciclovir has 8-20 times greater in vitro activity against CMV, is as active as acyclovir against HSV-1 and HSV-2, and is almost as active against VZV [37]. Ganciclovir exhibits excellent antiviral activity in tissue culture against CMV, HSV-1, HSV-2, EBV, VZV, and HHV-6 [36].

## Conclusions

Steroid refractory acute on chronic UC flares should be seen as a potential red flag for the investigation of CMV colitis with concomitant superinfection. UC patients with HSV and CMV dual infection should be initially treated with high-dose steroids to suppress the ongoing acute flair. Following a steroid prescription plan, it is appropriate to begin antiviral therapy to improve clinical symptoms, achieve remission at much higher rates, improve surgery-free survival, and offer long-term prophylaxis against reactivation.
